# Cortical thickness is related to working memory performance after non-invasive brain stimulation

**DOI:** 10.1590/1414-431X2023e12945

**Published:** 2023-10-20

**Authors:** L.B. Razza, M.A. Vanderhasselt, M.S. Luethi, J. Repple, G. Busatto, C.A. Buchpiguel, A.R. Brunoni, P.H.R. da Silva

**Affiliations:** 1 Department of Head and Skin - Psychiatry and Medical Psychology, Ghent University Hospital, Ghent University, Ghent, Belgium; 2 Ghent Experimental Psychiatry (GHEP) Lab, Ghent, Belgium; 3 Serviço Interdisciplinar de Neuromodulação, Laboratório de Neurociências (LIM-27), Departamento e Instituto de Psiquiatria, Hospital das Clínicas, Faculdade de Medicina, Universidade de São Paulo, São Paulo, SP, Brasil; 4 Department of Psychiatry, Psychosomatic Medicine and Psychotherapy, University Hospital, Goethe University, Frankfurt, Germany; 5 Institute for Translational Psychiatry, University of Münster, Münster, Germany; 6 Laboratório de Neuroimagem em Psiquiatria (LIM-21) e Instituto de Psiquiatria, Faculdade de Medicina, Universidade de São Paulo, São Paulo, SP, Brasil; 7 Divisão de Medicina Nuclear (LIM-43), Instituto de Radiologia, Hospital das Clínicas, Faculdade de Medicina, Universidade de São Paulo, São Paulo, SP, Brasil; 8 Departamento de Clínica Médica, Faculdade de Medicina, Universidade de São Paulo, São Paulo, SP, Brasil; 9 Hospital Universitário, Universidade de São Paulo, São Paulo, SP, Brasil

**Keywords:** Non-invasive brain stimulation, Cortical thickness, Individualization, Working memory, Voxel-based morphometry

## Abstract

Non-invasive brain stimulation (NIBS) probing the dorsolateral prefrontal cortex (DLPFC) has been shown to have little effect on working memory. The variability of NIBS responses might be explained by inter-subject brain anatomical variability. We investigated whether baseline cortical brain thickness of regions of interest was associated with working memory performance after NIBS by performing a secondary analysis of previously published research. Structural magnetic resonance imaging data were analyzed from healthy subjects who received transcranial direct current stimulation (tDCS), intermittent theta-burst stimulation (iTBS), and placebo. Twenty-two participants were randomly assigned to receive all the interventions in a random order. The working memory task was conducted after the end of each NIBS session. Regions of interest were the bilateral DLPFC, medial prefrontal cortex, and posterior cingulate cortex. Overall, 66 NIBS sessions were performed. Findings revealed a negative significant association between cortical thickness of the bilateral dorsolateral prefrontal cortex and reaction time for both tDCS (left: P=0.045, right: P=0.037) and iTBS (left: P=0.007, right: P=0.007) compared to placebo. A significant positive association was found for iTBS and posterior cingulate cortex (P=0.03). No association was found for accuracy. Our findings provide the first evidence that individual cortical thickness of healthy subjects might be associated with working memory performance following different NIBS interventions. Therefore, cortical thickness could explain - to some extent - the heterogeneous effects of NIBS probing the DLPFC.

## Introduction

Working memory is a cognitive system that allows the temporary storage and online manipulation of information that enables goal-directed behavior (1). Working memory is crucial in diverse cognitive functions, such as decision-making, learning, language, and reasoning, and plays an important role in our daily activities. Working memory declines with aging and deficits can be observed in several neuropsychiatric disorders, including schizophrenia, depression, and obsessive-compulsive disorder (2,3). The neurobiological underpinnings of working memory have been extensively investigated in healthy and patient populations in recent years (4,5). Neuroimaging investigations have consistently shown that non-emotional working memory tasks (n-back) are associated with the activation of the dorsolateral portion of the prefrontal cortex (DLPFC), the medial prefrontal cortex (mPFC), and the posterior cingulate cortex (PCC) - regions involved in the frontoparietal and default mode networks, which are involved in a series of cognitive process (6,7).

Since the DLPFC is easily reached compared to deeper cortical regions, a large number of studies using non-invasive brain stimulation (NIBS) interventions, especially transcranial direct current stimulation (tDCS) and repetitive transcranial magnetic stimulation (rTMS), have probed this area to modulate working memory in both healthy and neuropsychiatric participants (8,9). tDCS injects a weak electric current into the brain via electrodes placed on the scalp. The technique does not generate action potentials alone but can modulate brain activity towards an increase or decrease in endogenous neuronal firing (10). In turn, rTMS protocols can induce neuronal depolarization via focal electric currents applied to the brain, being able to increase or decrease neural activity (11). Initial studies have shown a potential increase in working memory performance after a course of rTMS and tDCS (12), but more recent studies presented a high variability (including null to small effects) of NIBS interventions when investigating working memory performance compared to placebo (13,14). A previous study of our team also corroborates these findings, reporting a null effect of prefrontal tDCS factor and a small positive effect of intermittent theta-burst (iTBS, a form of rTMS intervention) factor on reaction time of a working memory task in healthy subjects compared to placebo.

Heterogeneous effects of NIBS on the DLPFC on working memory performance might be explained by a variability of NIBS parameters and also by individual differences, such as anatomical variability (15). In terms of the former, individual anatomical brain biomarkers, including cortical volume and cortical thickness, were, respectively, found to be related to NIBS response in depression (16,17) and for tDCS in decision-making processes (18). Some studies with rTMS also showed that changes in longitudinal cortical thickness are associated with rTMS responders in depression (19). So far, however, there are no studies assessing the role of baseline individual brain anatomy of healthy volunteers in predicting working memory performance following different NIBS interventions, even though previous research suggests an association between working memory performance and cortical thickness (20,21).

Therefore, based on our prior research (22), we proposed to investigate whether individual baseline cortical thicknesses of regions of interest (ROIs) in the brain are associated with working memory performance of healthy individuals submitted to tDCS, iTBS, and placebo in a within-subject design. Cortical thickness was chosen as the outcome as a previous study showed that it can account for a high variance in tDCS response (18). Based on previous studies (6,7), the bilateral DLPFC, mPFC, and PCC were selected as ROIs. We hypothesized that individual cortical thickness variability is associated with different working memory performance following tDCS and iTBS, which does not occur in the placebo group.

## Material and Methods

This study was based on data from a previous trial that measured working memory performance of healthy subjects after a course of NIBS protocols. The original study used a factorial, double-blinded, within-subjects design, in which participants were allocated to receive four different NIBS interventions (tDCS, iTBS, combined tDCS+iTBS, and placebo) on different days, in a randomized order (22) (the study design can be visually checked in the Supplementary Figure S1). The focus of our study was to investigate only the results of the tDCS, iTBS, and placebo groups, as the combined protocol is a novel intervention used only twice over the DLPFC, and our main objective was to investigate the impact of within-individual variability in more standard NIBS techniques. However, the results of the combined protocol are reported as a side analysis in the Supplementary Material.

### Participants

In our previous study (22), 24 healthy subjects with a mean age of 28.7 years (standard deviation (SD)=6.95) were included. They were right-handed volunteers, aged 18 to 45 years, both sexes, without neuropsychiatric disorders and/or clinical diseases. Participants were prescreened by e-mail and those who met inclusion criteria underwent on-site screening by a trained psychologist to check for previous or current psychiatric diagnoses based on the Diagnostic and Statistical Manual of Mental Disorders, Fifth Edition (DSM-5) (23), the Hamilton Depression Rating Scale (HDRS), the Beck Depression Inventory (BDI), and the Positive and Negative Affect Schedule (PANAS) scales. Exclusion criteria were specific contraindications to NIBS interventions and MRI (e.g., metal implants), habitual smoking (>10 cigarettes/day) or abuse/dependence on other drugs, pregnancy, and use of psychoactive drugs (including antidepressant drugs, benzodiazepines, and Z-drugs).

### Procedure

First, an anatomical T1-weighted imaging of the brain was performed using a 3T magnetic resonance imaging (MRI) scanner (General Electric PET/MRI equipment, USA), followed by a real-time MRI-guided neuro-navigation system (Brainsight, Rogue Resolutions, Inc., Canada) using the T1-weighted image to target the left and right DLPFCs (MNI152 stereotaxic coordinates, -38, +44, +26 and +38, +44, +26, respectively) (24). The experimental session was composed of prior baseline measurements (HDRS and BDI), followed by the NIBS sessions. The working memory task was assessed immediately after the end of the stimulation session.

### Back task

The 2-back task was programmed in E-prime 2.0 software (Psychology Software, Tools Inc., USA). The visual stimulus consisted of letters (A to Z) that appeared in a pseudo-randomized order on a computer screen of 15 inches. The applied protocol consisted of three blocks of 30 letters. Letters were displayed on the screen for 500 ms, with an interval of 3000 ms between displays. Each block consisted of 10 ‘target' letters, representing a total of 30 ‘targets'. Targets were letters identical to the ones presented two steps earlier in the trial sequence. Participants were instructed to press different keys on the keyboard for target (key ‘2') and non-target (key ‘0') stimuli. A brief practice containing 20 stimuli was conducted prior to the task. The 2-back task was chosen because it was previously associated with working memory improvement after NIBS in healthy participants (12).

### NIBS protocols

Based on previous studies investigating cognitive performance after tDCS in healthy volunteers (25), electrodes were positioned over the left (anode) and right (cathode) DLPFC located via neuronavigation, pointing towards ‘Cz'. TDCS was applied with a current of 2 mA through saline-soaked sponges of 25 cm^2^ and lasted 20 min (Neuroconn DC-Stimulator, Germany). Placebo tDCS used the same montage but delivered only an active current of 30 s on the beginning of the tDCS session (22).

iTBS protocol used a TMS coil applied with an angle of 45 degrees relative to the midline. The protocol consisted of 54 cycles of 10 triplet bursts with a train duration of 2 s and an interval of 8 s between trains (1620 pulses) at 110% of the resting motor threshold. The protocol lasted 8 min and 40 s. The coil B65 Active/Placebo MagVenture (Denmark) was used for both active and placebo protocols, as it has two identical sides for delivering active or placebo stimulation depending on the randomized codes imputed on the device (22).

### MRI acquisition

All structural brain MRIs were acquired in a 3-Tesla MR system (General Electric PET/MRI equipment, USA). Volumetric images were based on T1-weighted sequences using a 3D fast-field echo pulse sequence with the following parameters: field of view (FOV) of 25.6, time of repetition (TR) of 7.7 ms, time of echo (TE) of 3.1 ms, and 202 slices.

### Neuroimaging processing and cortical thickness quantification

The T1-weighted image of each participant was processed using the CAT12 toolbox (26) within the SPM12 software using MATLAB (UK). We performed a voxel-based processing for voxel-based morphometry (VBM), followed by a surface-based processing for surface-based morphometry (SBM), and finally a region-based processing for region-based morphometry (RBM). The VBM analysis incorporates tissue segmentation, spatial registration, adjustments for volume changes due to registration (modulation), as well as a convolution with a Gaussian kernel matrix (spatial smoothing with an 8-mm full-width to half-maximum (FWHM) filter). The latter steps are followed by the SBM, which also incorporates several different steps such as surface creation, surface registration, and spatial smoothing (applied using a 15 mm FWHM filter). In the surface creation step, a projection-based thickness method estimates both initial cortical thickness and central surface considering partial volume information, sulcal blurring, and asymmetries (26). The cortical thickness measurement captures the width of the gray matter band as the distance between its inner and outer boundaries in thousands of points. Then, topological correction is performed with spherical harmonics, followed by a surface refinement, resulting in the final central, pial, and white surface meshes (27). The pial and white matter surfaces are used to refine the initial cortical thickness using the FreeSurfer thickness metric (27). Finally, data was visually inspected.

In the RBM step, the Destrieux atlas (28) was used to fit individual surfaces using the spherical registration parameters determined during surface-based processing. Cortical thickness was then calculated for each ROI in native space.

**Figure 1:**
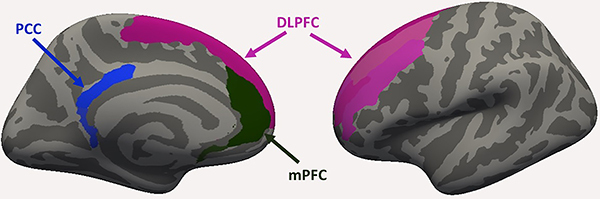
Brain regions of interest investigated in this study. PCC: posterior cingulate cortex; DLPFC: dorsolateral prefrontal cortex; mPFC: medial prefrontal cortex.

According to our *a priori* hypothesis, the investigated ROIs were the DLPFC, the mPFC, and the PCC of both hemispheres, and subregions of the Destrieux atlas were used to parse each of these ROIs. The DLPFC was composed of the superior and middle frontal gyrus and sulcus, the mPFC was composed of the anterior cingulate gyrus and sulcus and the subcallosal gyrus. Finally, the PCC was composed of its posterior ventral and posterior dorsal portions (Figure 1). The thickness of the ROI was obtained by the average of the sum of all subregions included in each ROI.

### Statistical analysis

Statistical analyses were performed using R version 4.1.2 (USA). Reaction time (in milliseconds (ms)) and accuracy (binary outcome) of the target stimuli were the dependent variables, while ROI thickness and protocol were the independent variables. Missed responses were considered errors, and reaction times <200 ms and >2500 ms were not considered genuine responses and were excluded (29). The preprocessing regarding the working memory task was identical to what was done in our previous study (22). Due to the non-normal distribution of reaction time responses (Supplementary Figure S2), generalized linear mixed models (GLMM; using the ‘lme4' package) with an inverse-Gaussian error distribution and an inverse link function were used. For accuracy, GLMMs with binomial distributions were employed. All models used the interaction between ROI thickness and protocol and were controlled for session (the order in which the different NIBS protocols were performed). The variable subject was included as a random intercept. Analyses were performed using the ROIs of both hemispheres, resulting in a total of six models for each outcome. All models were controlled for the effects of age and gender.

Secondly, as a supplementary analysis, we investigated the association between the working memory performance after the combined protocol only and cortical thickness using the same GLMM structures described above.

All results were considered significant at a P threshold of 0.05. As the analyzed brain regions were hypothesis-driven, we did correct for multiple testing, similar to what was done previously (16,30,31).

## Results

Our prior study included a sample of 24 healthy subjects, two of which were excluded because the T1-image of one subject did not pass the quality check and one subject did not receive the placebo stimulation. Therefore, 22 subjects were included in this study, presenting a mean age of 28.4 years (SD=7.1 years), mean education of 17 years (SD=3 years), and 77% were women. All the 22 participants underwent sessions of tDCS, iTBS, and placebo in a randomized order, with a total of 66 NIBS sessions being performed. Working memory performance per condition can be found in Supplementary Table S1.

### Working memory and ROI thickness

First, we evaluated whether there was an association between working memory performance in the placebo group and cortical thickness, but no significant association was found (Table 1).

**Table 1:** Association of working memory performance and cortical thickness in the placebo group.

ROI	Coefficient	SE	Statistic	P
Reaction time				
Left mPFC	-0.03	0.13	-0.24	0.811
Right mPFC	-0.04	0.12	-0.31	0.757
Left DLPFC	-0.06	0.12	-0.48	0.63
Right DLPFC	-0.04	0.12	-0.29	0.768
Left PCC	0.03	0.11	0.27	0.791
Right PCC	-0.12	0.14	-0.84	0.398
Accuracy				
Left mPFC	0.12	0.33	0.36	0.722
Right mPFC	0.07	0.31	0.22	0.828
Left DLPFC	-0.10	0.29	-0.36	0.717
Right DLPFC	-0.36	0.29	-1.25	0.211
Left PCC	-0.05	0.29	-0.18	0.858
Right PCC	-0.14	0.32	-0.44	0.66

For each region of interest (ROI), a generalized linear mixed models (GLMM) with an inverse-Gaussian error distribution and an inverse link function (same parameters as for main analyses) was set up with reaction time as outcome variable, and a logistic regression was performed with accuracy as the outcome variable. ROI and session were included as fixed effects and subject as a random intercept. The statistical parameters are *t*-values for GLMMS and z-scores for logistic regressions. DLPFC: dorsolateral prefrontal cortex; mPFC: medial prefrontal cortex; PCC: posterior cingulate cortex; SE: standard error.

**Figure 2:**
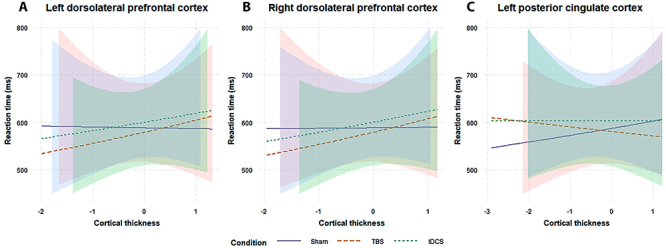
Association between baseline cortical thickness and working memory reaction time following non-invasive brain stimulation protocols. TBS: theta-burst stimulation; tDCS: direct current stimulation.

Therefore, we investigated the association between working memory performance after iTBS and tDCS compared to placebo and cortical thickness differences. Regarding reaction time, results revealed an inverse association between working memory performance following iTBS and tDCS (*vs* placebo) and cortical thickness in the right (coef=0.07, standard error (SE)=0.03, P-value=0.007 and coef=0.06, SE=0.03, P-value=0.037, respectively) and the left DLPFC (coef=0.08, SE=0.03, P-value=0.007 and coef=0.05, SE=0.03, P-value=0.045, respectively). In other words, right and left thinner DLPFC regions were associated with reaction time improvement (Table 2; Figure 2; Supplementary Figures S3 and S4). The findings also revealed that the thicker left PCC was associated with reaction time improvement after iTBS (coef=-0.07, SE=0.03, P-value=0.027). No association was found for the mPFC.

**Table 2:** Association between cortical thicknesses of regions of interest in the brain and working memory performance.

ROI	thCS				iTBS			
	Coefficient	SE	Statistic	P	Coefficient	SE	Statistic	P
Reaction time								
Left DLPFC	0.05	0.03	2.0	0.045*	0.070	0.03	2.70	0.007**
Right DLPFC	0.06	0.03	2.1	0.037*	0.070	0.03	2.70	0.007**
Left mPFC	-0.02	0.03	-0.74	0.457	0.008	0.03	0.39	0.750
Right mPFC	0.00	0.03	0.01	0.990	0.040	0.03	1.44	0.140
Left PCC	-0.04	0.03	-1.46	0.143	-0.070	0.03	-2.21	0.027*
Right PCC	0.04	0.03	1.55	0.121	0.040	0.03	1.35	0.178
Accuracy								
Left DLPFC	-0.16	0.14	-1.10	0.270	-0.140	0.14	-0.97	0.330
Right DLPFC	-0.08	0.15	-0.55	0.582	-0.030	0.15	-0.18	0.857
Left mPFC	0.17	0.18	0.91	0.362	0.030	0.19	0.13	0.896
Right mPFC	-0.04	0.19	-0.22	0.826	-0.070	0.20	-0.34	0.730
Left PCC	0.05	0.14	0.35	0.728	-0.020	0.15	-0.10	0.920
Right PCC	-0.11	0.17	-0.69	0.489	0.090	0.17	0.51	0.609

Statistics: *P<0.05 and **P<0.01; *t*-values for reaction time (generalized linear mixed models) or Z-scores for accuracy (logistic regressions). DLPFC: dorsolateral prefrontal cortex; mPFC: medial prefrontal cortex; PCC: posterior cingulate cortex; ROI: region of interest; SE: standard error; tDCS: transcranial direct current stimulation; iTBS: intermittent theta-burst stimulation.

No significant association between accuracy and ROI thickness was found (Table 2). Moreover, demographic variables did not influence the overall results (Supplementary Table S2).

The results from the association between cortical thickness and working memory performance after the combined protocol can be found in Supplementary Table S3.

## Discussion

In this study, based on data from our previous trial (22), we investigated whether individual brain cortical thickness of regions involved in working memory processes were associated with working memory performance of healthy subjects submitted to NIBS protocols. To the best of our knowledge, this is the first placebo-controlled study that investigated brain cortical thickness and working memory outcomes of tDCS and iTBS. All individuals received tDCS, iTBS, and placebo in a random order on different days (one week apart), so every subject served as its own control. Our findings revealed that cortical thickness of the bilateral DLPFC and left PCC was associated with reaction time performance after tDCS and iTBS sessions. No association was found for accuracy.

As expected, our results showed that inter-individual anatomical differences were associated with NIBS effects. The findings revealed that subjects with thinner DLPFC regions showed relatively faster reaction times than individuals with greater cortical thickness after tDCS and iTBS interventions compared to placebo, whereas the PCC, which was the deepest area investigated, presented a positive association between its left portion and reaction time performance after iTBS.

Interestingly, in our first study (22), no association was found between reaction time and tDCS, but only iTBS. We posit that the effects of tDCS in our previous study might have been mitigated by inter-individual variability when grouped working memory scores were considered in the analysis. Therefore, the present results add to the growing body of evidence emphasizing that the heterogeneous (or null) effects of tDCS might be due to individual anatomical differences, such as in previous studies (16,32). In turn, while the iTBS effects might also be dependent on individual variability, its stronger effects can also be found in a group-level analysis.

The inverse association between cortical thickness and working memory performance was already reported in previous studies using interventions other than NIBS. For instance, a recent trial investigated the changes in cortical thickness of healthy volunteers after two months of working memory training and found that some structural changes were negatively associated with cognitive performance, demonstrating that larger thickness reductions led to larger training-related behavioral improvements (33). The authors suggested that plastic changes may have occurred at a level of brain network in participants who underwent working memory training compared to placebo. Based on a previous study with TMS (34), we inferred that the negative association between working memory performance and cortical thickness of the DLPFC of healthy subjects might also be explained by compensatory effects of NIBS in regions with smaller neural populations.

Our results also showed that iTBS performance, but not tDCS, presented an association with the PCC, the deepest cortical thickness region investigated. As tDCS delivers a weaker electric current compared to iTBS and does not produce action potentials per se, we hypothesized that tDCS effects may have not been robust enough to modulate inner cortical structures compared to placebo, whereas iTBS might have been able to reach the PCC after only one active session. Based on these findings, we can assume that the PCC plays an important role in working memory processes and that iTBS mechanisms of action on the left DLPFC are associated with network-level efforts.

Moreover, we were not able to find an association between individual cortical thickness of the mPFC and working memory performance. Although preliminary studies suggest that bilateral tDCS montages over the DLPFC can induce stronger electric fields in the medial part of the PFC (35,36), this association has only been shown by computational modeling studies. Therefore, future studies are needed to investigate the combination of individual electric field strength in the mPFC and the working memory performance following tDCS.

Although our results provide the first evidence of the association between individual cortical thickness differences and working memory performance following an NIBS intervention, previous studies already suggested that brain anatomy might have an influence on NIBS response. For instance, a recent study showed that individual gray matter volume of the left DLPFC might be associated with tDCS antidepressant effects (16). Similar results were also found in studies using rTMS (37). Moreover, a study investigating the association between cortical thickness in the right prefrontal hemisphere and decision making performance of tDCS of healthy subjects (18) showed that individual cortical morphology of the targeted area accounted for almost 35% of the variance in cognitive performance across subjects. Taken together, the results presented here reinforced the need for studies with larger sample sizes aiming to evaluate the impact of individual cortical thickness or anatomical brain measures in the variability of NIBS responses over the DLPFC.

### Limitations

Our study has several limitations that should be discussed. First, the sample size was small. Thus, some analyses might have been underpowered, and future studies should use these results as hypothesis-driven. Second, other anatomical measures such as cortical volume could have been used for this study. However, as a previous study suggests that cortical thickness could be associated with tDCS response, and other studies with elderly show that cortical thickness is more sensitive to investigate response confounders (i.e., age and sex) (38), we considered this outcome more appropriate for the aim of this study. Third, we performed only a ROI-based analysis, therefore other regions that could be associated with working memory performance and NIBS were included in this analysis. Fourth, the working memory paradigm applied in this study (2-back) might not have been challenging enough for the study population, which had a high education level.

### Conclusion

This study provided initial evidence that individual differences in baseline cortical thickness of the DLPFCs targeted with tDCS and iTBS and deeper regions targeted with iTBS might be associated with reaction time performance. However, no association was found between accuracy and cortical thickness. The findings of our study can be useful to guide future studies investigating individual predictors of tDCS and iTBS probing the DLPFC for working memory performance.

## Supplementary Material

Click here to view [pdf].
